# High Seroprevalence of SARS-CoV-2 among Healthcare Workers in a North Italy Hospital

**DOI:** 10.3390/ijerph18073343

**Published:** 2021-03-24

**Authors:** Chiara Airoldi, Filippo Patrucco, Fulvia Milano, Daniela Alessi, Andrea Sarro, Maicol Andrea Rossi, Tiziana Cena, Silvio Borrè, Fabrizio Faggiano

**Affiliations:** 1 Department of Translational Medicine, Università degli Studi del Piemonte Orientale, 28100 Novara, Italy; andrea.sarro@uniupo.it (A.S.); fabrizio.faggiano@uniupo.it (F.F.); 2 Osservatorio Epidemiologico, 13100 Vercelli, Italy; filippo.patrucco@gmail.com (F.P.); fulvia.milano@uniupo.it (F.M.); daniela.alessi@aslvc.piemonte.it (D.A.); 20034828@studenti.uniupo.it (M.A.R.); tiziana.cena@med.uniupo.it (T.C.); silvio.borre@aslvc.piemonte.it (S.B.)

**Keywords:** seroprevalence, healthcare workers, SARS-CoV-2

## Abstract

Background: Healthcare workers (HCWs) have been the key players in the fight against the coronavirus disease 2019 (COVID-19) pandemic. The aim of our study was to evaluate the seroprevalence of severe acute respiratory syndrome coronavirus 2 (SARS-CoV-2) IgG anti-bodies. Methods: We conducted a cross-sectional study among workers of two hospitals and Territorial Medical and Administrative services in Northern Italy. From 8 May to 3 June 2020, 2252 subjects were tested. Seroprevalence and 95% confidence interval (CI) were calculated for all individuals who were stratified by job title, COVID-19 risk of exposure, direct contact with patients, unit ward, and intensity of care. Results: Median age was 50 years, and 72% of subjects were female. The overall seroprevalence was 17.11% [95% CI 15.55–18.67]. Around 20% of healthcare assistants were seropositive, followed by physicians and nurses (16.89% and 15.84%, respectively). HCWs with high risk of exposure to COVID-19 were more frequently seropositive (28.52%) with respect to those with medium and low risks (16.71% and 12.76%, respectively). Moreover, personnel in direct contact had higher prevalence (18.32%) compared to those who did not (10.66%). Furthermore, the IgG were more frequently detected among personnel of one hospital (19.43%). Conclusion: The high seroprevalence observed can be partially explained by the timing and the population seroprevalence; the study was conducted in an area with huge spread of the infection.

## 1. Introduction

Severe acute respiratory syndrome coronavirus 2 (SARS-CoV-2) is responsible for the coronavirus disease 2019 (COVID-19) pandemic, affecting millions of individuals worldwide (according to the WHO). In Italy, the outbreak’s results are constantly being updated, and the real prevalence of SARS-CoV-2 infection is ever-changing [1]. Currently, the diagnosis of COVID-19 is based on the detection of SARS-CoV-2 RNA via real-time reverse transcription polymerase chain reaction (RT-PCR) on nasopharyngeal swabs (NP). Nevertheless, serological tests to detect anti-SARS-CoV-2 antibodies were used in order to improve the knowledge on infection epidemiologic spread and to estimate the burden of this viral infectious disease [2].

Healthcare workers (HCWs) have been the key players in the fight against the COVID-19 pandemic, having placed themselves at high risk of catching SARS-CoV-2. A recent meta-analysis estimated that the overall proportion of HCWs testing SARS-CoV-2-positive (*n* = 12,901) was 8.7% (95% confidence interval: 6.7–10.9) among all enrolled subjects (*n* = 127,480). Furthermore, several studies reported the values of seroprevalence among HCWs, ranging from 0% to 45.3% [3]. Differences could be justified by settings, observation period, and strategies adopted by the government with the aim of reducing viral transmission (e.g., lockdown, quarantine measures, etc.) [4].

In Italy, a seroprevalence survey involving 64,660 people was conducted between 25 May and 15 July. This cross-sectional study reported an overall seroprevalence of 2.5%, even if there is a high variability among regions, with the highest results in Lombardia—in this region, 7.5% of positivity was reached [5]. Moreover, peak of 9.8% in Northern Italian regions was observed among HCWs. Additionally, Italian studies conducted in a specific hospital in Northern Italy showed the percentage of seropositivity to be around 5 to 7% [6,7,8,9], in line with national seroprevalence. Only one study conducted in Lodi hospital reported higher values (16.8%) [10].

The aim of the present study was to evaluate the seroprevalence of SARS-CoV-2 IgG anti-bodies among the personnel of Local Health Service (LHS) of Vercelli, which includes two hospitals and Territorial Medical and Administrative services. Moreover, we assessed if seroprevalence was influenced by job title, COVID-19 exposure risk, contact with patients, unit wards, and intensity of care. 

## 2. Materials and Methods

From 8 May to 3 June 2020, all personnel in LHS Vercelli were invited to test for SARS-CoV-2 serology as the Italian Region Council decided to organize a seroprevalence screening among all the healthcare workers. The only exclusion criterion was the absence of patient’s consent. The tested population included HCWs as well as technical and administrative staff; we decided to also include in our study workers without a permanent employment. LHS Vercelli encompasses 2 main hospitals (Vercelli “Sant’Andrea” Hospital and Borgosesia “Santi Pietro e Paolo” Hospital), as well as Territorial Medical and Administrative services. 

For each subject, we reported the following data derived from up-to-date administrative databases: demographics, occupation, and hospital and service where they worked (e.g., ward, ambulatory, laboratory, administrative). We also reported results from serological SARS-CoV-2 test and RT-PCR on NP performed before and after serological SARS-CoV-2 test results. 

Subjects were screened using The Liaison DiaSorin SARS-CoV-2 S1/S2 IgG test (DiaSorin, Saluggia, Italy). This is a fully automated quantitative serology test performed to detect solution for the detection of IgG antibodies against virus on a peripheral blood sample. The detection of neutralizing antibodies has 94.4% positive agreement to the Plaque Reduction Neutralization Test (PRNT) and sensitivity and specificity are 97.9 and 98.5, respectively. Positive or negative results were established by the following cuts off: <12 AU/mL: negative; ≥15AU/mL: positive. Moreover, the DiaSorin Molucular SimplexaTM COVID-19 Direct real-time RT-pCR assay was used for the in vitro qualitative detection of nucleic acid from severe acute respiratory syndrome coronavirus 2 in nasal swab specimens. Negative results do not preclude SARS-CoV-2 infection. 

To stratify seroprevalence proportion according to different levels of COVID-19 exposure and intensity of care, we categorized the unit wards into different groups by 2 physician connoisseurs of the hospital organization. On the basis of risk of exposure, we considered

(i) high risk: personnel in contact with patients diagnosed with COVID-19;
(ii) medium risk: personnel in contact with patients without test for SARS-CoV-2 or waiting for the result of tests, and personnel that work in wards where the patients do not wear continuously the mask due to the need to undergo a particular medical procedure;
(iii) low risk: the remaining personnel, including administrative staff.

On the basis of patients’ treatment, we identified four areas:

(i) high–intermediate intensity (high-flow oxygen therapy, continuous positive airway pressure, high-flow nasal cannula, non-invasive or invasive mechanical ventilation);
(ii) low intensity (room air or low flow oxygen therapy);
(iii) personnel in contact with outpatients;
(iv) administrative personnel.

Descriptive statistical analyses were conducted separately for the results of the serological test. We reported frequency and percentages (%), mean and standard deviations (SD), or median and interquartile range (IQR) for categorical and continuous variables. To compare associations between test positivity and variables, we performed chi-squared/Fisher’s test or Student’s *t*-test/non parametric test. The seroprevalence 95% confidence intervals (95% CI) were also calculated for all subjects and separately for the job title, the COVID-19 risk exposure, direct contact with patients, unit wards, and intensity of care, when data were available.

Moreover, to evaluate which covariates were associated with high seroprevalence, we performed logistic models and estimated odds ratios (ORs) and 95% CIs. In detail, to avoid the problem of sparse data, we aggregated some categories with few subjects. In addition, in order to evaluate the presence of multicollinearity, we performed the LASSO (least absolute shrinkage and selection operator regression) model and removed regression coefficients that were co-dependent. Then, we conducted different univariable models and each variable with *p*-value less than 0.10 was considered for the multivariable model. The final multivariable model was obtained using backward procedure with a threshold of 0.10, including age and sex independently from their statistical significance as they were considered of clinical interest. 

Significance level was set generally at *p* < 0.05. Data were analyzed using SAS 9.4 (SAS Institute, Cary, NC, USA).

The study was approved by the Ethical Committee of our institution and was conducted in accordance with the Declaration of Helsinki.

## 3. Results

In our analysis, we included 2252 subjects; among them, 1750 (77.71%) had a permanent employment. Two subjects were excluded from our study because serological results were doubtful. Median age was 50 years [IQR 18–39] and the majority (72%, N = 1636) were female.

Overall, 385 subjects showed anti-SARS-CoV-2 IgG, and the seroprevalence was 17.11% [95% CI 15.55–18.67]. The anti-SARS-CoV-2 IgG titer in the population who were seropositive (*n* = 385) had a median of 50.80 UA/mL [IQR 25.50–98.70] and reached a maximum of 919 UA/mL. 

Demographic and occupational characteristics are shown in Table 1, both with a more detailed focus on subjects with permanent contract (*n* = 1749). Moreover, seroprevalences and 95% CIs for each level of variables are reported. 

Higher seroprevalence was observed among healthcare assistants with a value of 20.65%, followed by physicians and nurses (16.89% and 15.84%, respectively). On the contrary, lower values of positive seroprevalence (8.57%) were observed for administrative staff. HCWs exposed to high risk of COVID-19 were more frequently seropositive (28.52%) in contrast with those with medium and low risks (16.71% and 12.76%, respectively). Moreover, personnel in direct contact with patients’ care showed higher prevalence (18.32%) compared to those who were not (10.66%). Ward units were significantly associated with positive results, even though the higher seroprevalence was found among Vercelli Hospital laboratory personnel (20.72%). Moreover, some clusters were observed in territorial pharmaceutical services, where all subjects were seropositive (8/8, 100%), in surgery department (25/34, 75.53%), in urology (7/12, 58.33%), and in pulmonology (17/35, 48.57%). In addition, 53.33% (16/30) and 44.58% (37/83) of HCWs respectively in orthopedics and general internal medicine tested positive. Nevertheless, in the emergency and infectious disease departments, the estimated prevalence was lower: 15.71% (11/70) and 7.41% (2/27), respectively. 

Generally, the IgG were more frequently detected among personnel of Vercelli Hospital (19.43%, 95% CI 17.31–21.69) compared to Borgosesia Hospital (7.36%, 95% CI 4.78–9.94). In addition, while significant differences of seroprevalence were found among wards and services in Vercelli (*p*-value <0.001), the hospital of Borgosesia seemed to have a less heterogeneous seroprevalence (*p*-value 0.2621). 

Intensity of care was related to the seropositivity prevalence—in particular, those subjects who were involved with high–intermediate levels of care (high flow oxygen therapy, continuous positive airways pressure, high flow nasal cannula, non-invasive or invasive mechanical ventilation) revealed a higher prevalence of seropositivity (26.90%) compared with low intensity (room air or low flow oxygen therapy) (16.22%), outpatient care (10.75%), or administrative personnel (10.78%). 

Moreover, the prevalence was 16.18% [95% CI 14.45–17.91] and 20.36% [95% CI 16.83–23.89] for permanent and non-permanent workers, respectively.

Then, we collected results of 758 and 495 NP swab tests performed before and after serological evaluation, respectively. Reasons why workers were subjected to NP swab were different (e.g., contact with COVID-19 subjects, symptoms) and were not recorded. However, subjects who had a previous NP swab were significantly associated with higher seroprevalence: 59.22% vs. 28.42% in subjects with and without anti-SARS-CoV-2 IgG, respectively (*p* < 0.0001). Moreover, 44.74% of positive subjects had a prior positive NP swab, whereas 1.70% seronegative subjects had a prior positive NP swab. Interestingly, in these nine subjects, specific serological response was not detectable, despite the time elapsed from NP and serological test ranges from 46.37 and 57.37 days, or some errors in the test occurred (false negative). (Table 2)

Among subjects who underwent NP swab test after serologic evaluation (*n* = 495), 9 positive NP swabs were found: 8 among seropositive subjects and 1 among seronegative ones (*p* = 0.0347). For the eight positive subjects, the NP were performed in a median time of 13.52 [IQR 12.42–21.54] days within serological results, while for the subject with negative results, 39 days had passed.

Finally, the univariable and multivariable models were performed, and results are reported in Table 3. No variables were excluded as no multicollinearity problem was identified by the LASSO model. Despite all covariates, except age and sex, being significantly associated with seroprevelance (*p*-value <0.0001) in univariable models, direct contact with patients lost its significance (*p*-value 0.3671) in multivariable logistics. Interestingly, the higher OR (4.80; 95% CI 1.12–20.53) was estimated for subjects who worked in Vercelli Hospital (ref Territorial Service). Moreover, people who worked in low-intensity care (room air or low flow oxygen therapy) had a threefold increased risk (OR) in comparison with administrative staff; in addition, the risk was higher than the estimates in high–intermediate intensity (OR 1.19). Workers at medium COVID-19 exposure risk had an OR of 1.63 [95% CI 1.07–2.47], which was also higher than people at high risk (1.07, 95% CI 0.65–1.76), (ref: low risk). In terms of job title, the estimates in multivariable models were weaker (closer to 1) than the univariable ones; however, healthcare assistants were related to a doubled risk (ref: administrative).

To better understand the role of hospitals in the SARS-CoV-2 diffusion, we implemented separate models for the two hospitals; no analysis was conducted on territorial services due to the low number of subjects (*n* = 58) (Table A1 in Appendix A). In Vercelli Hospital, job title and intensity of care were statistically associated with seroprevalence. To illustrate this, we calculated an OR of 2.03 [95% CI 1.06–3.89] for healthcare assistants (ref: administrative staff) and 3.60 [95%CI 2.30–5.62] for low intensity, similar to the main analysis. However, when the analysis was repeated for Borgosesia, it was not possible to detect any difference (*p*-value > 0.10) among groups in univariable models, and thus multivariable logistic was not performed.

## 4. Discussion

In this hospital-wide screening study for SARS-CoV-2 seroprevalence among the entire staff of two hospitals involved in COVID-19 patients’ care, we observed an overall prevalence of 17.11%, with higher positivity proportion among HCWs. Interestingly, we found that important differences among hospitals (19.43% vs. 7.36%) were observed and possible clusters of cases into specific unit wards were identified. Moreover, there were significant differences of seroprevalence among intensity of care, job title, and COVID-19 exposure risk. Our study revealed a higher risk in people who worked in room air or low-flow oxygen therapy and in healthcare assistants. Finally, we suspected that, given the little experience in dealing with the new virus, workers without enough clinical preparation and knowledge in infectious disease were more affected. 

Similar seroprevalence was observed in studies conducted in China (17.14%) and England (17.97%), encompassing 105 and 6440 HCWs, respectively [11,12]. Both studies were performed in a similar time period to our analysis, as they had a lag of some months from the SARS-CoV-2 spread in the two countries; the first was conducted at the end of January, while the second in April–June. Nevertheless, seroprevalence in our study was higher than those reported by the Italian seroprevalence surveys for comparable group (9.8% in North Italy region) and than those conducted on some Italian hospitals: 4.56% [7], 5.13% [8], 6.93% [9], and 7.43% [6], while our results are in line with Baracco et al. (16.79%) [10]. The test used in these studies had similar characteristics in terms of sensitivity and specificity, with diagnostic accuracy measures higher than 90%. Differences could be first explained by different timing; aforementioned observational studies with lower prevalence were conducted before (April–May), while Baracco et al. performed his analysis between May and June [10]. Moreover, geographical gradient and different organizational approaches adopted could be suspected. For example, in the Veneto region, Plebani et al. [7] observed a lower prevalence, probably due to a different public health organization—this region implemented a broad community-based strategy that relied on its more robust public health network and local integration of services. On the contrary, Piedmont based the care model on a large hospitalization relying mainly on its network of public and private hospital structures. Furthermore, in our study, we observed an increased risk for Vercelli Hospital compared to Borgosesia Hospital. In the first part of the first wave of the epidemic, Borgosesia Hospital was protected from the first insurgence of the epidemic because the main strain of COVID-19 patients was deviated toward Vercelli. Once COVID-19 patients began to be hospitalized in Borgosesia, the personnel were probably better prepared, as well as better equipped with appropriate personal protective equipment, in order to tackle the impact of the emergency and to prevent hospital transmission. 

Not surprisingly, we observed differences in seroprevalence according to job role categorization. Although the majority of the literature [5,6,7,8,9,13] showed a lower prevalence among employees or administrative staff, some studies have reported inverse results [14,15]. The higher value of seropositivity among healthcare assistants was observed in Baracco et al.’s study (22.18%) [10], followed by nurse and medical staff, with similar levels of prevalence (17.95% and 17.03%, respectively). Coherent percentages were also reported by Rudberg et al. [13], with 25.47% among assisting nurses, 21.86% among nurses, and 19.13% among physicians. Although results of Plebali et al. were generally lower [7], a similar trend was observed. These can be explained by the fact that HCWs, in particular nurses and healthcare assistants, spent a lot of time in contact with patients COVID-19 in order to carry out their assistance and they are the first at bed-site in case of any patient need [16]. Moreover, the higher risk in non-medical categories could be explained by the lack of consolidated hygiene habits, especially for those working in services that do not generally come into contact with outpatients and visitors. We revealed, on the contrary, a lower seroprevalence among workers in administration, probably due to organizational strategies adopted during the first phase of the pandemic such as smart-working, social distancing, and no front office activities. 

In addition, in our study, higher percentages of seropositivity were found among subjects that worked in areas of higher intensity of care for COVID-19; specifically, the prevalence was higher in wards and services where patients were treated with respiratory support (i.e., CPAP, NIV, or IMV), which could generate aerosol droplets contaminated with viral particles [17]. Nevertheless, these results are in contrast with Grant et al.; the authors found that seropositivity was lower among intensive care unit HCWs [18]. Several reasons could explain this finding such as the enhanced personal protective equipment for intensive care unit HCWs; the fact that the intubated patients are ventilated on a closed circuit; and the fact that COVID-19 patients, who require admission on an intensive care unit, are often admitted around day 10 of the natural history of their illness, by which point viral loads of patients tend to decrease [3]. 

No consideration can be made for subjects with permanent and non-permanent contracts, as the second group included HCWs recruited as a component of the task force, both with precarious administrative staff. Furthermore, we were not able to analyze in detail the data on NP swabs as they were available only for a non-random sample of people. However, we found that a previous positive PCR test increases the probability of a positive SARS-CoV-2 antibodies test in HCWs, as suggested by some literature [19,20].

Our study has several limitations. First, only 82.24% of LHS Vercelli workers with permanent contract were submitted to serology; however, even if we were not able to identify factors related to the adherence, the percentage of participation was sufficiently high to consider the results valid. Second, the population included in the study was representative of the staff working in a middle-sized healthcare organization that included both hospitals and territorial services, and cannot be considered representative of the whole Italian population at the time of the study. Finally, we did not collect symptoms mentioned by the study population, with this potentially limiting the real incidence and prevalence of COVID-19. 

## 5. Conclusions

In conclusion, wide screening of seroprevalence and its implementation with RT-PCR test are needed in order to understand some risk factors associated with diffusion of SARS-CoV-2 and may encourage future infection control and occupational health practices.

## Figures and Tables

**Table 1:** Demographic and occupational characteristics of the 2250 workers in terms of serological test results. Absolute and percentage frequencies are reported.

		**Serological Test**		
**Variable**	**Total (N = 2250)**	**Negative (N = 1865)**	**Positive (N = 385)**	**Seroprevalence [95% CI]**
**Sex**				
Female	1636 (72.71)	1347 (72.23)	289 (75.06)	17.67 [15.85–19.60]
Male	614 (27.29)	518 (27.77)	96 (24.94)	15.64 [12.85–18.75]
**Age (years)**				
*<30*	249 (11.07)	212 (11.37)	37 (9.61)	14.86 [10.68–19.90]
*30–39*	333 (14.80)	274 (14.69)	59 (15.32)	17.72 [13.77–22.25]
*40–49*	504 (22.40)	414 (22.20)	90 (23.38)	17.86 [14.61–21.49]
*50–59*	842 (37.42)	717 (38.45)	125 (32.47)	14.85 [12.51–17.43]
*60+*	322 (14.31)	248 (13.30)	74 (19.22)	22.98 [18.50–27.97]
		**Serological Test**		
	**Total (N = 1749)**	**Negative (N = 1466)**	**Positive (N = 283)**	**Seroprevalence [95% CI]**
**Job title**				
Nurses/physiotherapist	688 (39.34)	579 (39.50)	109 (38.52)	15.84 [13.11–18.57]
Physicians	296 (16.92)	246 (16.78)	50 (17.67)	16.89 [12.62–21.16]
Healthcare assistants	465 (25.69)	369 (25.17)	96 (33.92)	20.65 [16.97–24.32]
Administrative staff	175 (10.01)	160 (10.91)	15 (5.30)	8.57 [4.42–12.72]
Technical staff	118 (6.75)	106 (7.23)	12 (4.24)	10.17 [4.72–15.62]
Other	7 (0.40)	6 (0.41)	1 (0.35)	14.29 [0.4–57.87]
**COVID-19 exposure risk**				
Low	1005 (63.18)	964 (65.76)	141 (49.82)	12.76 [10.79–14.73]
Medium	353 (20.18)	294 (20.05)	59 (20.85)	16.71 [12.82–20.61]
High	291 (16.64)	208 (14.19)	83 (29.33)	28.52 [23.33–33.71]
**Direct contact with patients**				
No	488 (27.90)	436 (29.74)	52 (18.37)	10.66 [7.92–13.39]
Yes	1261 (72.10)	1030 (70.26)	231 (81.63)	18.32 [16.18–20.45]
**Unit wards**				
Vercelli—ward	587 (33.56)	434 (29.60)	153 (54.06)	26.06 [22.51–29.62]
Vercelli—outpatient facility	214 (12.24)	185 (12.62)	29 (10.25)	13.55 [8.97–18.14]
Vercelli—laboratories	111 (6.35)	88 (6.00)	23 (8.13)	20.72 [13.18–28.26]
Vercelli—administrative	385 (22.01)	338 (23.06)	47 (16.61)	12.21 [8.94–15.48]
Borgosesia—ward	230 (13.15)	208 (14.19)	22 (7.77)	9.57 [5.76–13.37]
Borgosesia—outpatient facility	80 (4.57)	77 (5.25)	3 (1.06)	3.75 [0–7.91]
Borgosesia—laboratories	41 (2.34)	39 (2.66)	2 (0.71)	4.88 [0–11.47]
Borgosesia—administrative	43 (2.46)	41 (2.80)	2 (0.71)	4.65 [0–10.95]
Territorial services	58 (3.32)	56 (3.82)	2 (0.71)	3.45 [0–8.14]
**Intensity of Care**				
Administrative personnel	464 (26.53)	414 (28.24)	50 (17.67)	10.78 [7.95–13.60]
Contact with outpatients	400 (22.87)	357 (24.35)	43 (15.19)	10.75 [7.71–13.79]
Low intensity	450 (25.73)	377 (25.72)	73 (25.80)	16.22 [12.82–19.63]
High–intermediate intensity	435 (24.87)	318 (21.69)	117 (41.34)	26.90 [22.73–31.06]

**Table 2:** Results of healthcare workers (HCWs) with previous nasopharyngeal swabs (N = 758). *p*-values were obtained using chi squared/Fisher’s test or Student’s *t*-test/non parametric test, as appropriate.

		Serological Test		
Variable	Total (N = 758)	Negative (N = 530)	Positive (N = 228)	*p*-Values
NP swab				
Negative	647 (85.36)	521 (98.30)	126 (55.26)	<0.0001
Positive	111 (14.64)	9 (1.70)	102 (44.74)	
Time from NS to ST				
Median [IQR], days	34.46 [18.45–49.35]	29.63 [16.37–45.38]	44.36 [30.38–56.42]	<0.0001

**Table 3:** Univariable (*) and multivariable (**) logistic models (outcome = seroprevalence). Odds ratios and 95% confidence intervals were reported. ^#^ indicates statistically significant association with *p*-value less than 0.05.

Variable	OR * [95% CI]	OR ** [95% CI]
**Sex**		
Male	1	1
Female	1.19 [0.87–1.61]	1.29 [0.92–1.80]
**Age (years)**		
<30	1	1
30–39	0.74 [0.40–1.36]	0.71 [0.37–1.35]
40–49	0.76 [0.43–1.33]	0.83 [0.45–1.50]
50–59	0.57 [0.33–0.98] ^#^	0.71 [0.40–1.27]
60+	0.75 [0.41–1.39]	1.06 [0.54–2.08]
**Job title**		
Administrative staff	1	1
Nurses/physiotherapist	2.01 [1.14–3.54] ^#^	1.28 [0.68–2.42]
Physicians	2.17 [1.18–3.99] ^#^	1.36 [0.68–2.68]
Healthcare assistants	2.78 [1.56–4.93] ^#^	1.93 [1.03–3.62] ^#^
Other	1.24 [0.57–2.71]	1.11 [0.50–2.48]
**COVID-19 exposure risk**		
Low	1	1
Medium	1.38 [0.99–1.91]	1.63 [1.07–2.47] ^#^
High	2.73 [2.00–3.72] ^#^	1.07 [0.65–1.76]
**Direct contact with patients**		
No	1	1
Yes	1.88 [1.36–2.59] ^#^	-
**Hospital**		
Territorial services	1	1
Vercelli	6.75 [1.64–27.85] ^#^	4.80 [1.12–20.53] ^#^
Borgosesia	2.22 [0.52–9.58]	1.19 [0.26–5.34]
**Intensity of care**		
Administrative	1	1
Contact with outpatients	1.00 [0.65–1.54]	1.26 [0.78–2.03]
Low intensity	3.05 [2.12–4.38] ^#^	2.99 [1.71–5.24] ^#^
High–intermediate intensity	1.60 [1.09–2.36] ^#^	1.19 [0.73–1.95]

## Data Availability

Data are not available.

## Appendix A

**Table A1:** Univariable (*) and multivariable (**) logistic models (outcome = seroprevalence) separately for Vercelli and Borgosesia hospitals. Odds ratios and 95% confidence intervals are reported.

		Vercelli	
Variable	N = 1297	OR * [95% CI]	OR ** [95% CI]
**Sex**			
Male	340 (26.21)	1	1
Female	957 (73.79)	1.27 [0.92–1.76]	1.25 [0.87–1.78]
**Age (years)**			
<30	58 (4.47)	1	1
30–39	174 (13.42)	0.77 [0.39–1.54]	0.85 [0.41–1.75]
40–49	321 (24.75)	0.79 [0.41–1.50]	0.96 [0.49–1.87]
50–59	567 (43.72)	0.58 [0.31–1.08]	0.84 [0.44–1.61]
60+	177 (13.65)	0.73 [0.37–1.46]	1.24 [0.59–2.63]
**Job title**			
Administrative staff	146 (11.26)	1	1
Nurses/physiotherapist	468 (36.08)	2.37 [1.31–4.30]	1.22 [0.62–2.38]
Physicians	234 (18.04)	2.06 [1.08–3.93]	1.17 [0.57–2.41]
Healthcare assistants	341 (26.29)	3.33 [1.83–6.08]	2.03 [1.06–3.89]
Other	108 (8.33)	1.29 [0.58–2.87]	1.17 [0.52–2.65]
**COVID-19 exposure risk**			
Low	858 (66.15)	1	1
Medium	190 (14.65)	1.67 [1.13–2.46]	-
High	249 (19.20)	2.75 [1.99–3.81]	-
**Direct contact with patients**			
No	429 (33.08)	1	1
Yes	868 (66.92)	2.30 [1.65–3.21]	-
**Intensity of care**			
Administrative	411 (31.69)	1	1
Contact with outpatients	213 (16.42)	1.29 [0.79–2.09]	1.18 [0.71–1.99]
Low intensity	326 (25.13)	3.75 [2.56–5.47]	3.60 [2.30–5.62]
High–intermediate intensity	347 (26.75)	1.74 [1.16–2.61]	1.56 [0.99–2.45]
		**Borgosesia**	
**Variable**	**N = 394**	**OR [95% CI]**	**OR ** [95% CI]**
**Sex**			
Male	79 (20.05)	1	1
Female	315 (79.95)	1.22 [0.45–3.31]	-
**Age (years)**			
<30	27 (6.85)	1	1
30–39	59 (14.97)	0.42 [0.10–1.82]	-
40–49	96 (24.37)	0.45 [0.12–1.68]	-
50–59	167 (42.39)	0.37 [0.11–1.26]	-
60+	45 (11.42)	0.56 [0.13–2.46]	-
**Job title**			
Administrative staff	29 (7.36)	1	1
Nurses/physiotherapist	168 (42.64)	2.35 [0.30–18.67]	-
Physicians	59 (14.97)	4.39 [0.52–36.94]	-
Healthcare assistants	121 (30.71)	1.72 [0.20–14.55]	-
Other	17 (4.31)	4.39 [0.52–36.94]	-
**COVID-19 exposure risk**			
Low	190 (48.22)	1	1
Medium	162 (41.12)	1.78 [0.80–3.96]	-
High	42 (10.66)	0.81 [0.17–3.82]	-
**Direct contact with patients**			
No	56 (14.21)	1	1
Yes	338 (85.79)	2.34 [0.54–10.14]	-
**Intensity of care**			
Administrative	50 (12.69)	1	1
Contact with outpatients	132 (33.50)	1.97 [0.42–9.31]	-
Low intensity	109 (27.66)	2.16 [0.45–10.39]	-
High–intermediate intensity	103 (26.14)	2.02 [0.41–9.89]	-
